# Downregulation of VRK1 by p53 in Response to DNA Damage Is Mediated by the Autophagic Pathway

**DOI:** 10.1371/journal.pone.0017320

**Published:** 2011-02-28

**Authors:** Alberto Valbuena, Susana Castro-Obregón, Pedro A. Lazo

**Affiliations:** 1 Experimental Therapeutics and Translational Oncology Program, Instituto de Biología Molecular y Celular del Cáncer, Consejo Superior de Investigaciones Científicas (CSIC) - Universidad de Salamanca, Salamanca, Spain; 2 Department of Developmental Genetics and Molecular Physiology, Instituto de Biotecnología, Universidad Nacional Autónoma de México, Cuernavaca, Morelos, México; Wayne State University School of Medicine, United States of America

## Abstract

Human VRK1 induces a stabilization and accumulation of p53 by specific phosphorylation in Thr18. This p53 accumulation is reversed by its downregulation mediated by Hdm2, requiring a dephosphorylated p53 and therefore also needs the removal of VRK1 as stabilizer. This process requires export of VRK1 to the cytosol and is inhibited by leptomycin B. We have identified that downregulation of VRK1 protein levels requires DRAM expression, a p53-induced gene. DRAM is located in the endosomal-lysosomal compartment. Induction of DNA damage by UV, IR, etoposide and doxorubicin stabilizes p53 and induces DRAM expression, followed by VRK1 downregulation and a reduction in p53 Thr18 phosphorylation. DRAM expression is induced by wild-type p53, but not by common human p53 mutants, R175H, R248W and R273H. Overexpression of DRAM induces VRK1 downregulation and the opposite effect was observed by its knockdown. LC3 and p62 were also downregulated, like VRK1, in response to UV-induced DNA damage. The implication of the autophagic pathway was confirmed by its requirement for Beclin1. We propose a model with a double regulatory loop in response to DNA damage, the accumulated p53 is removed by induction of Hdm2 and degradation in the proteasome, and the p53-stabilizer VRK1 is eliminated by the induction of DRAM that leads to its lysosomal degradation in the autophagic pathway, and thus permitting p53 degradation by Hdm2. This VRK1 downregulation is necessary to modulate the block in cell cycle progression induced by p53 as part of its DNA damage response.

## Introduction

The cellular response to DNA damage is partly mediated by the p53 tumor suppressor, which determines the response specificity among different possibilities, such as cell cycle arrest, DNA repair, induction of apoptosis [1], [2] or autophagy [3], [4]. In cells responding to DNA damage, p53 has to be phosphorylated in its N-terminal transactivation domain, where several residues [5] are targeted by several kinases implicated in the response to different types of cellular damage or stress [6]. The consequence of these phosphorylations is to generate a transcriptionally active p53 protein, but differences in the pattern of multiphosphorylation can condition p53 protein interactions with transcriptional cofactors, and thus affect the specificity of the response [7], [8], [9]. The phosphorylation of p53 in Thr18 is the most critical phosphorylation for selective binding to transcriptional coactivators, such as p300, or preventing binding to negative regulators, such as Hdm2 [7], [8], [9]. To the specificity of these cofactor interactions also contribute phosphorylations in Ser15 or Ser20 [7], [8], [9]. The p53 molecule is stabilized by phosphorylation; and phosphorylated p53, which accumulates in response to DNA damage [5], cannot be degraded by the proteasome, because it cannot interact with mdm2/Hdm2 [7], [8], [9]. In this context p53 phosphorylation in Thr18 is the main switch from binding to mdm2 to interaction with transcriptional cofactors [7], [8], [9]. Biological responses mediated by p53 are a consequence of a complex network of positive and negative autoregulatory loops [10].

VRK1 (vaccinia-related kinase-1) is a novel ser-thr kinase that participates in cell cycle regulation [11], [12]. VRK1 is expressed in the G0 exit-G1 entry, behaving as an immediate-early gene like *MYC* and *FOS* [13], being expressed before cyclin D1 [13], and forming part of the *CCDN1* (*cyclin D1*) gene transcriptional complex [14]. VRK1 is also required for assembly of the nuclear envelope later in mitosis [15], [16], and is affected by its interaction with Ran, a protein regulating nuclear transport [17]. VRK1 knock-down induces a block in cell cycle progression [18] before the restriction point in G1 [13], resulting in a mitotic delay [13]. In head and neck carcinomas the VRK1 protein level correlates with cell proliferation markers, such as Ki67 [19]. One of the best characterized targets of VRK1 is p53, which is specifically phosphorylated in Thr18 [18], [20] disrupting the interaction with Hdm2, and leading to its accumulation [18]. But if p53 is maintained in a Thr18 phosphorylated form it cannot interact with Hdm2 [21], [22], and there would be a permanent cell cycle arrest and possibly cell death by apoptosis or autophagy. Thus a mechanism that downregulates the level of VRK1, a stabilizer of p53, to prevent a long-lasting p53 accumulation has been identified, which is not mediated by ubiquitylation, and that requires the lysosome [23], [24]. The mechanism that downregulates VRK1 is inducible by an accumulation of p53 after a stress response, and requires de novo transcription of a gene not yet identified but whose product targets VRK1 to enter the lysosomal pathway of protein degradation [23], [24]. By removing VRK1, p53 can be dephosphorylated and thus become accessible to Hdm2 and subsequent degradation in the proteasome. This mechanism is altered in lung carcinomas with p53 mutations, which have very high levels of VRK1 [25].

Among the degradation processes regulated by p53 is autophagy. By this process cells remove and digest endogenous proteins, particularly those that are very stable, functioning as an important mechanism for tissue remodeling [26] and maintaining cellular homeostasis [27], but it can also result in a form of cell death, thus having a dual role [28], [29]. In normal cells autophagy contributes to regulate basal levels of cytosolic and particulate proteins [3], a process that is further activated in response to several types of stress, including DNA damage. Autophagy is required for recycling of proteins implicated in negative cell cycle regulation, and can provide a survival strategy to tumor cells [26]. Recently a protein, DRAM (Damage-Regulated Autophagy Modulator), whose expression is induced by p53, has been shown to participate in degradation of stable proteins; DRAM ( is a novel damage-regulated autophagy modulator) component of the cell autophagic response [30]. Autophagy is partly regulated by p53-induced DRAM expression [30], and since p53-induced VRK1 degradation requires entry in the endosomal-lysosomal pathway and is mediated by an unknown gene [23], DRAM might be a likely candidate to participate in this process, since VRK1 is also a very stable protein [24]. In this work it has been tested the possibility that DRAM might be implicated in the autophagic degradation of VRK1 protein induced by p53 in the context of DNA damage response induced by UV light.

## Results

### DRAM gene expression is induced in response to DNA damage

**Figure 1:**
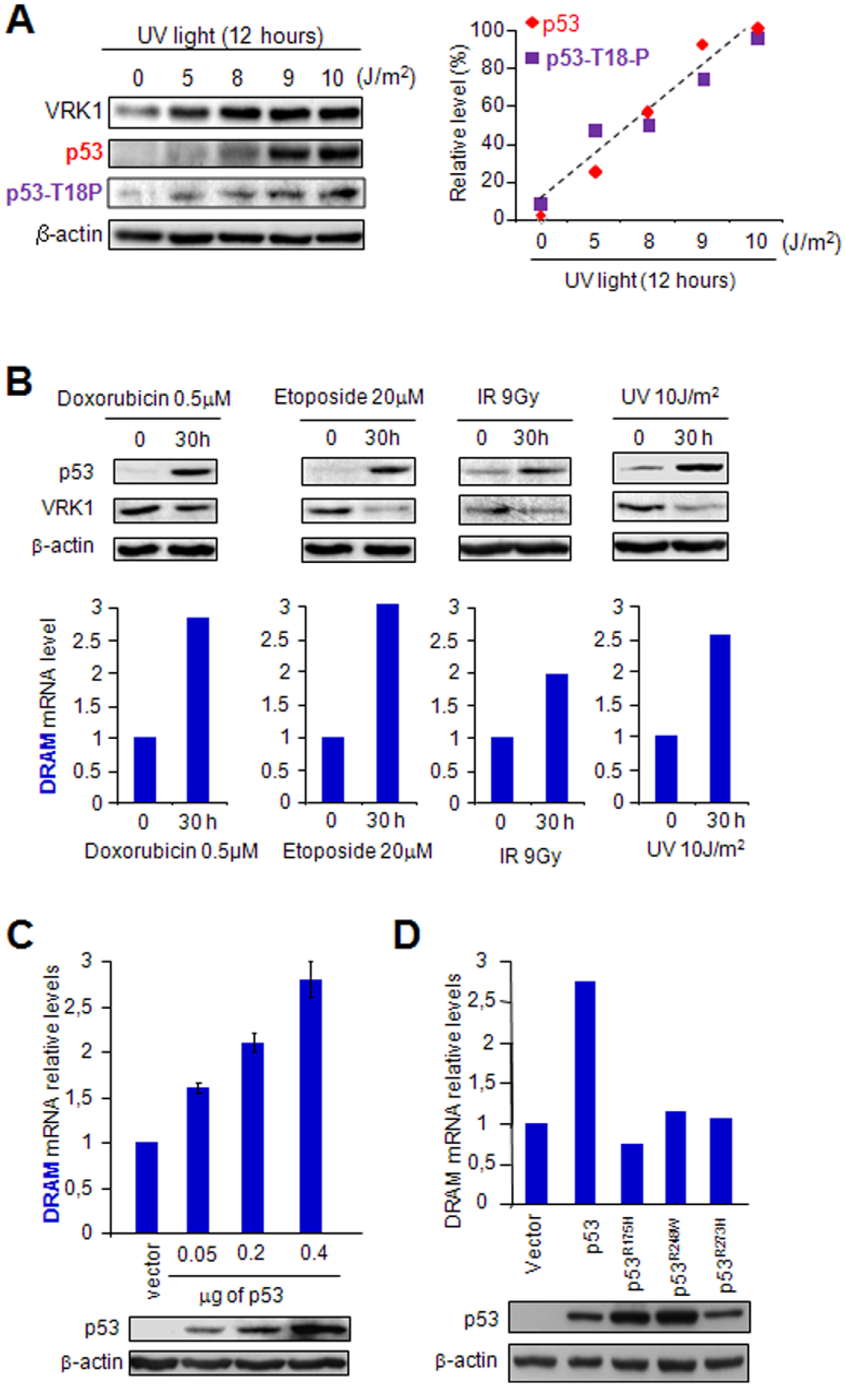
Effect of p53 on the transcription of endogenous DRAM gene. (A) Determination of the optimal dose of UV light that induces p53 stabilization and its phosphorylation in Thr18 in the WS1 cell line. To the right is shown the quantification of p53 and p53 phosphorylated in Thr18 as a function of the UV dose. (B) Different types of DNA damage induce endogenous p53 accumulation, and VRK1 downregulation in WS1 fibroblasts (p53+/+) determined by western blot (top). DNA damage also induces DRAM accumulation detected by qRT-PCR in human WS1 fibroblasts. The DNA damage agents used were doxorubicin, etoposide, ionizing radiation and UV-C light (254 nm). (C) H1299 (p53−/−) cells transfected with increasing amounts of plasmid pCB6+p53 and expression of *DRAM* was determined by qRT-PCR. Values are the mean of three experiments with standard deviation. Same amount of DNA was used in all transfections that were completed with empty vector as necessary. (D) H1299 (p53−/−) were transfected with the indicated plasmids pCB6+p53 (wt), pCMV-p53^R175H^, pCMV-p53^R248W^ and pCMV-p53^R273H^, and the effect on the expression of endogenous *DRAM* gene expression was determined by qRT-PCR. In the immunoblots (IB) at the bottom is shown the correct expression of the different p53 proteins, wild-type or mutants.

Different types of DNA damage can induce p53 phosphorylation and its accumulation [5]. *DRAM* gene induction by DNA damage and p53 accumulation was detected in RKO and Saos-2 cells [30]. Therefore, it was studied if in DNA damage responses, DRAM activation and mainly VRK1 downregulation were also detected in normal human WS1 fibroblasts that have a wild-type p53. For this aim WS1 cells were treated with several types of DNA damaging agents, such as ionizing radiation (IR) or ultraviolet-C light (254 nm) and also doxorubicin and etoposide, as positive controls. The dose of UV used was selected for its maximum effect on p53 accumulation and its phosphorylation in Thr18 (Fig. 1A). The time selected for observation was based on the timing of activation and transcriptional responses known to be mediated by p53 [5]. All these DNA damaging agents induced an accumulation of endogenous p53 protein and downregulation of VRK1 protein (Fig. 1B, top), as well as activation of *DRAM* gene expression (Fig. 1B, bottom), which was determined as positive internal control of the p53 response to DNA damage [30] in order to detect the relative change of VRK1 with respect to DRAM in the same cell line.

Next, it was tested if activation of endogenous *DRAM* gene expression has the same p53 transcriptional requirements as those required for VRK1 lysosomal proteolytic downregulation induced by p53. Downregulation of VRK1 induced by p53 requires de novo transcription of an unknown p53-induced gene, an effect that was not induced by the most common p53 mutations in humans, R175H, R248W and R273H [23]. For this aim, H1299 cells (p53−/−) were transfected with a wild-type p53 construct or the three most common p53 mutants detected in human cancer. Wild-type p53 induced *DRAM* gene expression in a p53 dose dependent manner (Fig. 1C), but none of the p53 mutants was able to activate *DRAM* gene expression (Fig. 1D). Thus the effect of p53 on *DRAM* expression is similar to those reported for induction of VRK1 protein downregulation [23], [25]. This suggested that DRAM might be a candidate protein, or a component of the route, required to mediate p53-dependent degradation of VRK1 in lysosomes.

### DRAM downregulates VRK1 protein level

Previously it has been ruled out the implication of the ubiquitin pathway. VRK1 is not ubiquitylated and its proteolytic p53-induced downregulation is not mediated by mdm2 [23]. VRK1 is also insensitive to mdm2 overexpression and it downregulation also occurs in mdm2−/− cells [23]. VRK1 protein kinase has been shown to be a new target of proteolytic degradation by the lysosomal pathway in a p53-dependent manner; VRK1 degradation is inhibited by lysosomal inhibitors, such as chloroquinne and leupeptin [23], [24]. The VRK1 protein has several target sequences for the endosomal-lysosomal pathway. There are two types of targeting motifs for this pathway: Sequences for endosomal targeting (End) and sequences for lysosomal-endosomal targeting (LysEnd). VRK1 protein contains both of these motifs [24]. The target sequences within VRK1 protein are located at 107–110, 126–129, 249–252 and 317–320 aminoacids for End sequences; and 191–196 and 304–309 aminoacids for LysEnd target sequences (ELM database).

**Figure 2:**
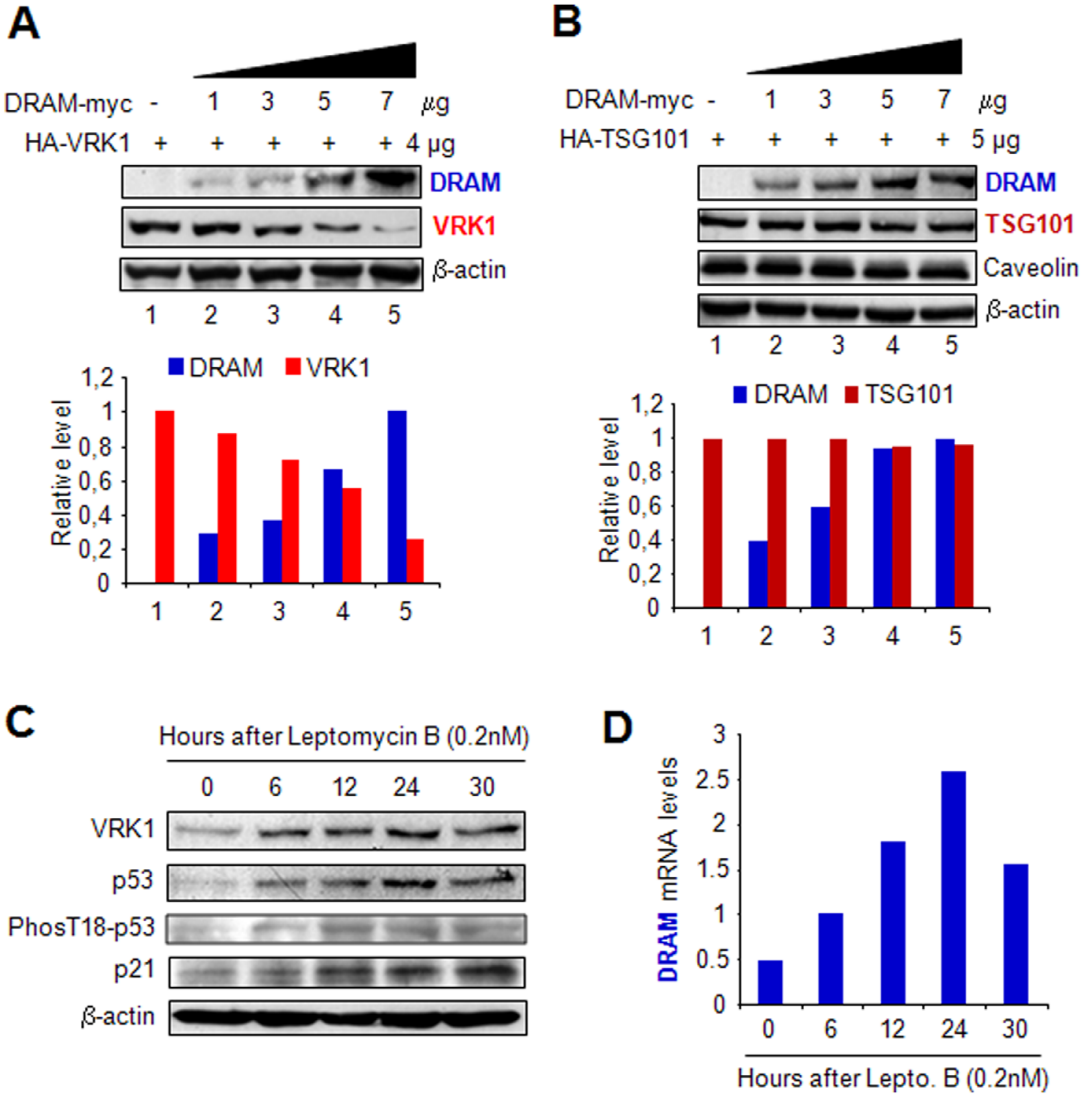
Dose dependent effect of DRAM on VRK1, and TSG101 protein levels. The variation in levels of VRK1 (A), and the control endosomal protein TSG101 implicated endocytic recycling of plasma membrane receptors and caveolin (B) were determined in response to increasing levels of DRAM. H1299 (p53−/−) cells were transfected with a fixed amount of VRK1 and TSG101 plasmids, and varying concentrations of DRAM. Plasmid pCDNA3-DRAM-Myc-His [30], plasmid pCEFL-HA-VRK1 [18], [23] and plasmid pHA-TSG101 [65] were used. To the bottom is shown the quantification of corresponding immunoblots in the linear response range. VRK1 and TSG101 proteins were detected with an anti HA antibody. DRAM was detected with an anti myc antibody. C. The block of nuclear export by leptomycin B in WS1 cell line prevented degradation of VRK1 even though there is an accumulation p53, which is also phosphorylated in Thr18 and induces *DRAM* gene expression. D. Accumulation of p53 by leptomycin B induces *DRAM* gene expression in WS1 cells as determined by quantitative qRT-PCR.

Therefore, it was studied if downregulation of VRK1 requires the participation of lysosomal DRAM protein, as a component of its degradation route, which by its characteristics is a candidate to mediate this effect [30]. The direct effect of DRAM protein on VRK1 level was determined. For these experiments the lung carcinoma H1299 (p53−/−) cell line that lacks p53 was used. This cell line was transfected with increasing levels of DRAM and studied its effect on VRK1 protein level and on TSG101 protein, which was used as a negative control for lack of effect on the promoter of their common expression vector. DRAM induced a dose dependent degradation of the VRK1 protein (Fig. 2A), but not of the TSG101control expressed from the same type of vector (Fig. 2B). TSG101 is an endosomal protein implicated in recycling of plasma membrane receptors and viral entry, thus since this protein was not affected it indicated that VRK1 and TSG101 must be in different types of endosomal vesicles [31], [32]. Caveolin a marker for a subtype of vesicles located in trans-Golgi and plasma membrane [33] was also not affected (Fig. 2B). These data suggested that there are two different types of endosomal vesicles, some regulating receptor recycling and others involved in autophagy that can be discriminated by DRAM. Also the effect of DRAM on the closely related VRK2A and VRK2B proteins [19], [34] was determined with a similar result (Fig. S1A, B). These results indicated that DRAM protein participates in an intermediate step required for VRK1 and VRK2 protein degradation by their entry in a subtype of endosomal vesicles destined for autophagy.

Most of the human VRK1 protein is located in the nucleus [18], but DRAM is acting in the cytosol. This implies that VRK1, in order to be degraded by this route, has to be transported to the cytosol. Degradation of VRK1 might occur by elimination of the cytosolic pool, which requires the export of nuclear VRK1 protein. VRK1 has a nuclear export signal in its C-terminal region (residues 298–310) [20]. To test this possibility, cells were treated with leptomycin B that blocks nuclear export [35]. Leptomycin B induced the accumulation of nuclear p53 [36] that is phosphorylated in Thr18 (Fig. 2C), and thus transcriptionally active which can induce two p53-dependent genes, p21 [36] and *DRAM* gene expression [37]. The induction of p21, a cell cycle inhibitor, was confirmed in an immunoblot (Fig. 2C). The DRAM expression is indeed induced by leptomycin in the WS1 cell line (Fig. 2D), but in this situation it does no degrade VRK1 (Fig. 2C) due to their nuclear localization. This result indicates that DRAM is participating in the removal of the cytosolic pool of VRK1.

The instability of VRK1 is not due to loss of its activity, since kinase-dead VRK1(K179E) is equally degraded by increased levels of p53 or DRAM (Fig. S1C).

### A subpopulation of DRAM colocalizes in Golgi apparatus and endosome vesicles with VRK1

**Figure 3:**
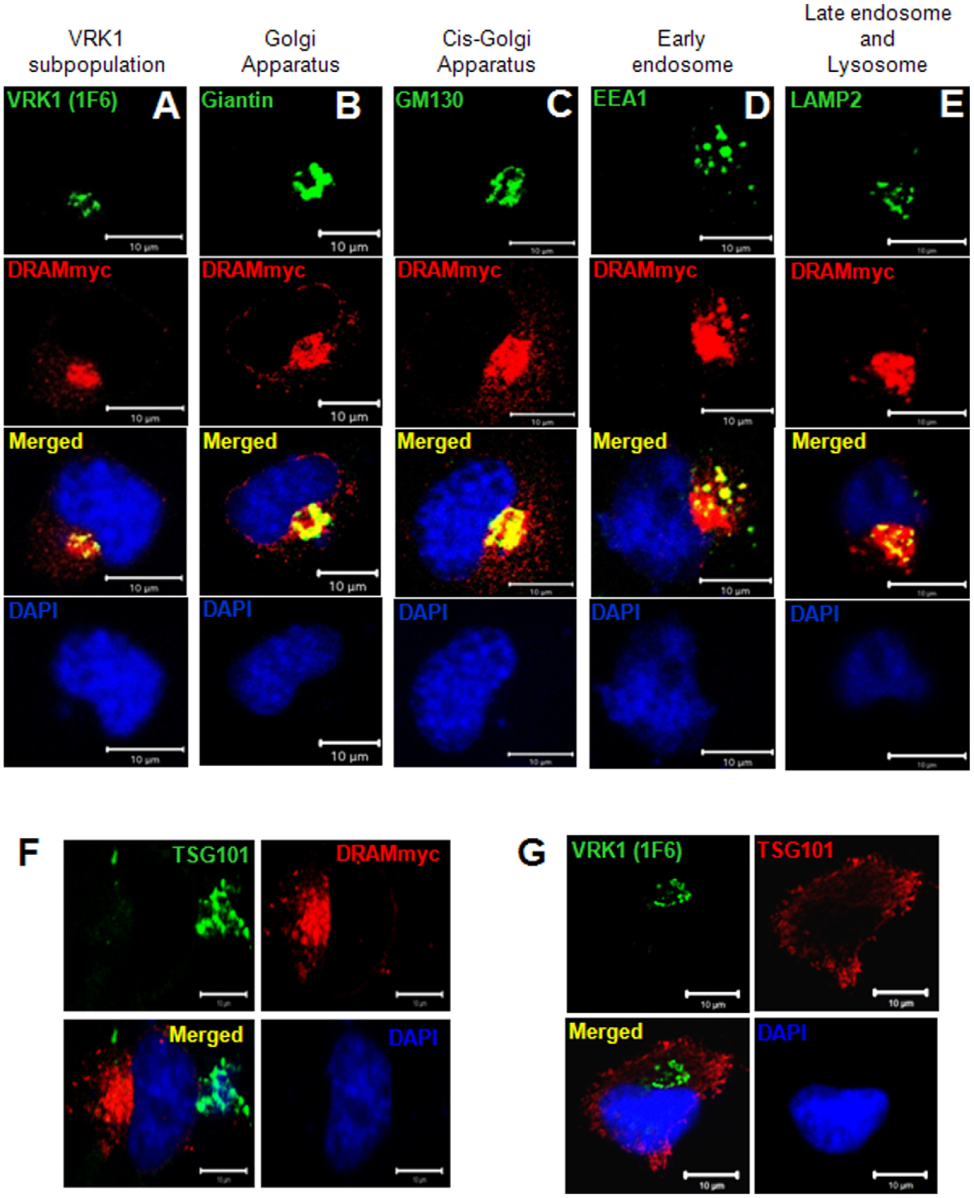
Subcellular localization of DRAM. (A–E) Colocalization of DRAM in different Golgi-endosome-lysosome compartments. (F) TSG101 and DRAM do not colocalize in endoplasmic vesicles. (G) TSG101 and VRK1 colocalize in different locations in the absence of DRAM. In these experiments, H1299 cells were transfected with plasmid pCDNA3-DRAM-Myc-His and plated on 10-cm^2^ dishes (5×10^5^) containing 1-cm-diameter sterile glass coverslips. The coverslips were stained twenty-four hours after DRAM transfection with specific antibodies for the endogenous proteins: VRK1 (1F6), giantin, GM130, EEA1 and LAMP2. DRAM was detected with an anti-myc epitope antibody. TSG101 was detected with a polyclonal antibody.

VRK1 is mostly nuclear, but there is always a subpopulation that is cycling in the cytosol [38] and can be detected in cytosolic vesicles, mainly in the Golgi apparatus [39], and can be detected with a specific antibody [38]. This VRK1 subpopulation enters a degradation pathway that ends in the lysosome [23]. Overexpressed DRAM was detected in lysosomes colocalizing with cathepsin D [40]. To ascertain if cytosolic VRK1 could also be detected in a common intracellular compartment with DRAM protein, the subcelular location of DRAM was determined in combination with several components of the Golgi-endosome-lysosome vesicular traffic that were used as markers. VRK1 is already known to be present in Golgi, colocalizing with giantin [38], [39]. DRAM protein was partially detected colocalizing with both VRK1 and giantin in cytosolic vesicles (Fig. 3A, B). DRAM also colocalized with GM130 (Fig. 3C), a marker of cis-Golgi; with EEA1, a marker for early endosome vesicles (Fig. 3D) and with LAMP2, a lysosomal marker (Fig. 3E). DRAM was detected in all these compartments suggesting that DRAM localization is more widely expressed than previously reported [30], and is not limited to lysosomes, but also colocalize with the Golgi as part of the degradation pathway of endosomal-lysosomal intracellular traffic.

DRAM might be a component of a specific subtype of endosomal vesicles required for lysosomal fusion, or represent a ligand for stable proteins that have to be removed. Therefore it was determined if a direct interaction between VRK1 and DRAM could be detected in reciprocal immunoprecipitation experiments. No direct interaction was detected (not shown), thus suggesting that DRAM is more likely to be a marker of the vesicle subtype and which is necessary for degradation. TSG101 is a protein implicated in endosomal traffic related to receptor recycling [31], [32], [41], therefore it was tested if TSG101 could intracellularly colocalize with DRAM (Fig. 3F). TSG101 and DRAM do not colocalize in intracellular vesicles indicating that they are markers for different subpopulations of endosomal vesicles. It was also determined the localization of VRK1 and TSG101 (Fig. 3G); these two proteins do not colocalize, but in the presence of low level of endogenous DRAM, TSG101 is more dispersed and some is located in the plasma membrane, its natural location before it is internalized.

### VRK1 downregulation after p53 stabilization by DNA damage follows DRAM accumulation

**Figure 4:**
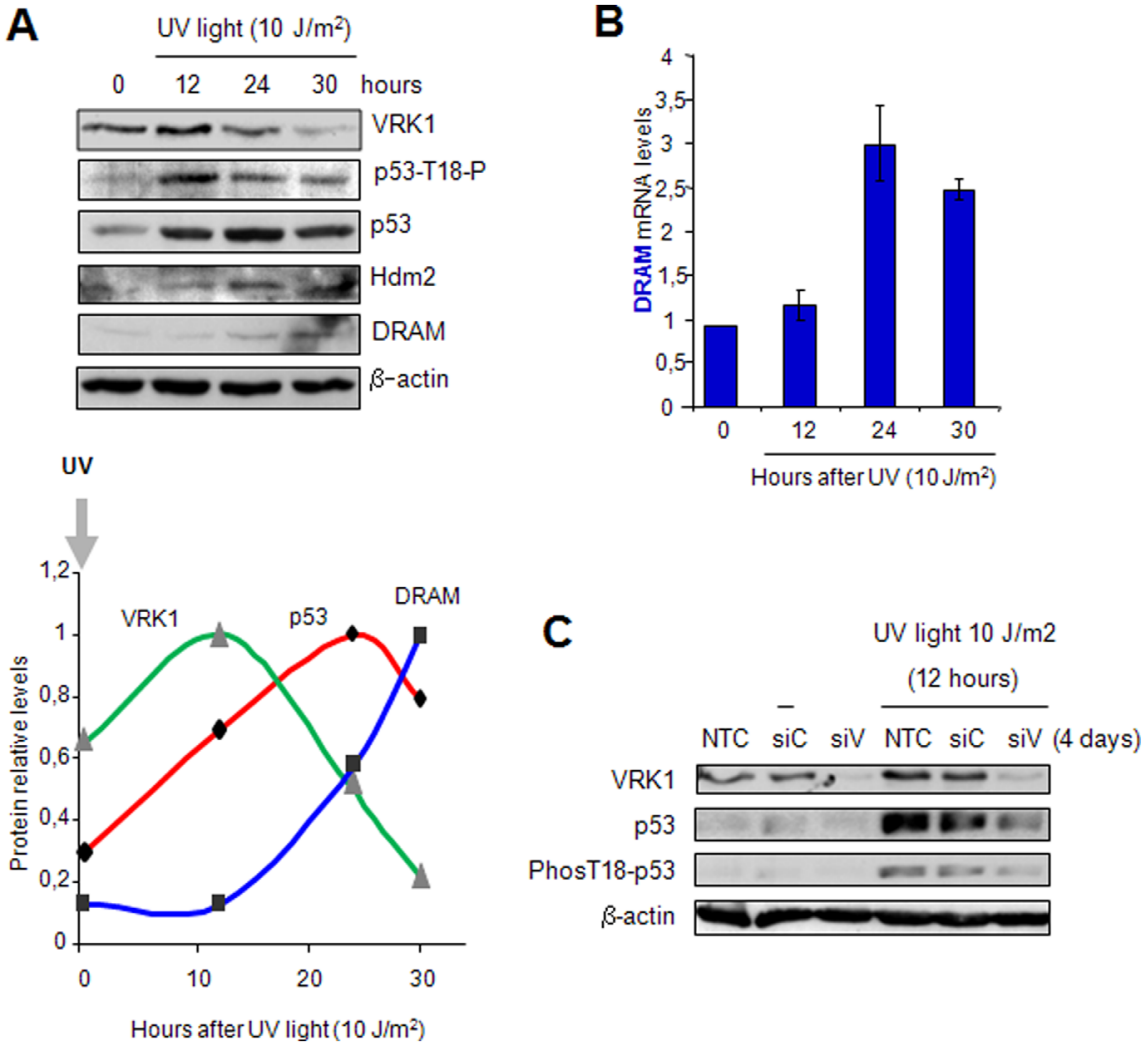
Sequential changes in VRK1, p53 and DRAM protein levels induced by UV in WS1 human fibroblasts. (A) Levels of the three proteins VRK1, p53 and DRAM after treating human fibroblasts WS1 with UV light. The relative level of each protein was quantified and represented in the graph at the bottom. (B) Quantification of DRAM RNA levels by qRT-PCR at different time points following treatment with UV light. Mean of three independents experiments with standard deviation. (C). Knockdown of VRK1 (siV), but not controls (siC and NTC), prevented the accumulation of p53 and its phosphorylation in Thr18 in response to UV irradiation. Knock-down siRNA transfections were performed 96 hours before the start of UV treatment. Cell lysates were prepared 12 hours after irradiation.

It was previously reported that VRK1 downregulation induced by p53 required de novo gene expression of an inducible gene that targets VRK1 for lysosomal degradation [23]. The contribution of ubiquitin ligases to VRK1 degradation has been rule out [23]. VRK1 is not ubiquitylated and VRK1 degradation is insensitive to mdm2 and to proteasome inhibitors, and VRK1 is also degraded in mdm2−/− cells [23]. One likely candidate is *DRAM*, a p53-induced gene [30], and the results above suggest that indeed this might be the case. In order to determine the potential relation between DRAM, VRK1 downregulation and p53 levels, a temporal assay of sequential changes in VRK1, p53 and DRAM protein levels was performed in WS1 cells after irradiation with UV-C light (254 nm). Normal human WS1 fibroblast cell line was treated with UV (10 J/m2) and the level of different proteins determined at different time points. Shortly after irradiation there is an accumulation of VRK1 and p53, followed by an accumulation of DRAM and Hdm2. As p53 increases so does the level of DRAM protein, which is followed by a reduction of VRK1 protein (Fig. 4A). This reduction in VRK1 is accompanied by a decrease in p53 phosphorylated in Thr18, and thus also the initiation of a reduction in total p53 protein due to its accessibility to the accumulated Hdm2 (Fig. 4A). The quantification of the blot is shown at the bottom (Fig. 4A). In this system the accumulation of DRAM protein (Fig. 4A) was a consequence of the activation of *DRAM* gene transcription as determined by qRT-PCR at different times after UV treatment (Fig. 4B).

Since p53 is stabilized by phosphorylation, we tested if downregulation of VRK1 would also prevent accumulation of p53 in response to UV treatment. Control cells accumulated p53 in response to UV, but the knockdown of VRK1 prevented the accumulation of p53, and its Thr18 phosphorylation induced by UV light with respect to the siControl and non-transfected control cells (Fig. 4C).

### Knockdown of DRAM and Beclin-1 prevents VRK1 downregulation induced by UV light

**Figure 5:**
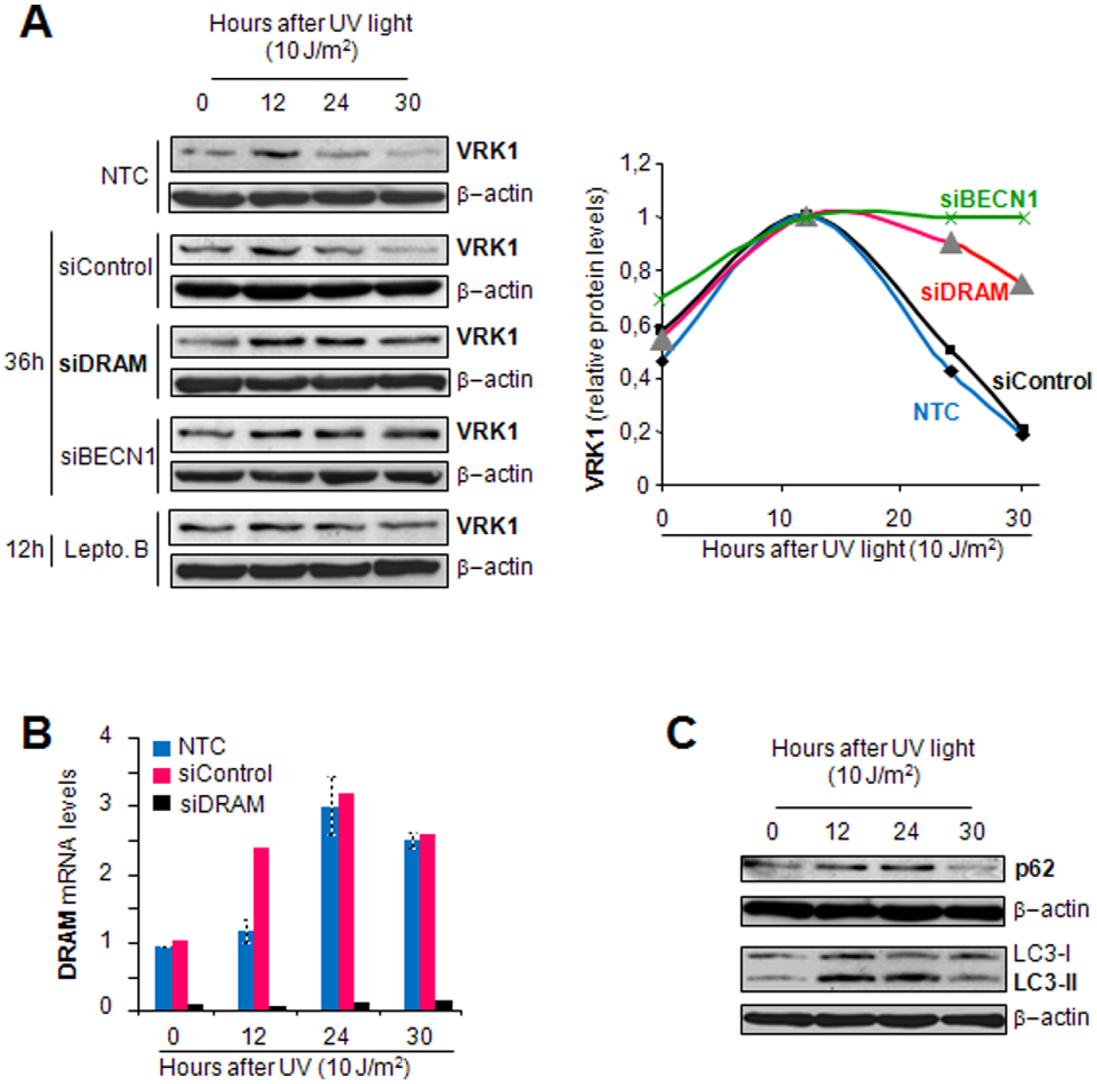
Knock-down of DRAM and Beclin-1 (BECN1), and addition of leptomycin B prevented downregulation of VRK1 induced by UV light. (A) Human fibroblast WS1 cells were transfected with siControl, siDRAM-01, siBECN1-smart pool, or treated with leptomycin B. After that, these cells were irradiated with UV-C light and the protein levels determined at different time points. The changes in levels of VRK1 protein were detected with the 1B5 mAb. The quantification of the blots is shown in the graph at the right. (B). The effectiveness of the DRAM knock-down was determined by qRT-PCR and the result shown in the bar graph at the bottom, and siBECN1 by western blot (Fig. S2). Knock-down siRNA transfections were performed 48 hours, and addition of leptomycin B was 12 hours, before the start of UV treatment. (C) P62/SQSTM1 and LC3B are proteins degraded by autophagy. P62/SQSTM1 and LC3B proteins are also degraded in response to UV light, following a transient accumulation in autophagosomes after induction of damage [60], [61].

To confirm the implication of DRAM in VRK1 downregulation induced by UV light, the level of endogenous DRAM was knocked-down by specific siRNA in WS1 fibroblasts. VRK1 protein level was higher in response to UV when DRAM was knocked-down (Fig. 5A). SiDRAM-01 was very effective in downregulating DRAM mRNA levels, while *DRAM* expression was induced in non-transfected or in siControl cells (Fig. 5B). These results confirmed the role of DRAM in UV-light induced downregulation of VRK1.

Beclin-1 is a protein that is required for autophagy [42], [43]. To further confirm that VRK1 degradation induced by UV is indeed entering the autophagic pathway, the effect of eliminating Beclin-1 was also determined. The knock-down of Beclin1 prevented UV induced degradation of VRK1, an effect that was also observed by either addition of leptomycin B or siDRAM, all of which prevented VRK1 protein degradation (Fig. 5A). These results confirmed that VRK1 degradation requires nuclear export to enter the autophagic degradation process. Leptomycin B is known to induce p21 to block cell cycle progression and DRAM in the autophagic pathway, both as a result of the nuclear accumulation of p53 [36], [37]. Beclin-1 knockdown by specific siRNA is effective and was not affected by UV irradiation (Fig. S2). In addition to the activation of p53 and the autophagic route in response to UV light, it was tested the effect of UV treatment on the level of p62/SQSTM1 and LC3B, two proteins known to be degraded by autophagy [44], [45], [46], and which were used a positive controls for autophagy in our system. Both, LC3 and p62/SQSTM1 (Fig. 5C) behave in a similar way as VRK1 (Fig. 5A) in response to DNA damage by UV light.

## Discussion

The downregulation of cellular proteins induced by p53 is a phenomenon that is lately acquiring relevance due to the role that p53 plays in several processes such as cell cycle, apoptosis and autophagy. However, the particular context that makes p53 to induce one biological response or another is not completely understood, although some of the components have already being identified. The determination of a specific response is likely to be dependent on protein levels, and the degree of phosphorylation of the individual components that can regulate interactions with co-transcriptional activators, which affect the selection of the genes regulated by p53. Among p53-regulated genes is *DRAM* [30] that is implicated in the removal of long-lived proteins [26]. One of these proteins is VRK1 that has a very large half-life [13].

**Figure 6:**
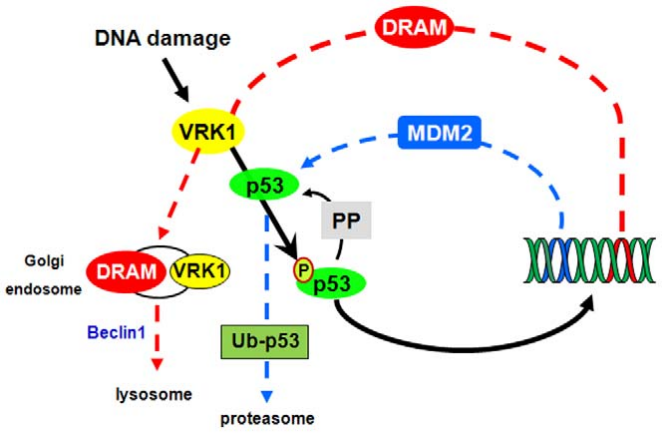
Model of the autoregulatory VRK1-p53-Hdm2-DRAM loop. Several types of DNA damage mechanisms can induce VRK1, stabilizing and activating p53-dependent transcription (black line). Among the p53-induced genes, Hdm2 promotes p53 degradation via ubiquitylation (blue line), and DRAM induces VRK1 degradation in the lysosome (red line). VRK1 and DRAM are in the same late endosomal vesicle that fuses to lysosomes, but do not interact directly. PP: unknown phosphatase.

Kinases that stabilize p53 are likely candidates to be selectively removed, or inactivated, in order to permit p53 dephosphorylation; making it accessible to Hdm2 and susceptible to ubiquitin-mediated downregulation, and thus generating fluctuations of their relative levels of expression [47], [48], which can result in regulatory loops [10]. In this context, the induction by p53 of DRAM protein, which contributes to the removal of VRK1 protein, is consistent with this picture (Fig. 6). Because VRK1 is a stabilizer and activator of p53 transcriptional activity, the downregulation of p53 levels requires the removal of its stabilizer, VRK1, otherwise the p53 phosphorylated in Thr18 cannot interact with Hdm2 [9] and will not be degraded. Thus, it is likely that the regulation of p53 is accompanied by an additional mechanism needed to downregulate its stabilizer kinase, VRK1, by a p53 dependent gene, as it is the case of *DRAM*. The double autoregulatory loop of VRK1 and p53, in which DRAM and Hdm2 participate is represented in Fig. 6. Briefly, the activation by phosphorylation of p53 in response to UV light induces an activation of gene transcription. But a permanent activation of p53 dependent transcription will result in either cell death or permanent cell cycle arrest. Therefore mechanisms to make transient this arrest are necessary at later times to prevent potentially deleterious effects. It is well known the autoregulation mediated by Hdm2 targeting p53 for degradation in the proteasome [49], and it has been detected in the case of other activating kinases such as ATM [50] and CHK2 [51], but this action requires a p53 induction of phosphatases, such as PPM1D [52] and Wip1 [51]. To these effects it has to be added the removal of VRK1 by DRAM. DRAM is also induced by UV light [37], an effect mediated by p53. These autoregulatory mechanisms can be partly implicated in the cellular fluctuations of p53 levels [53], [54], and should be integrated in the complex regulation of p53 [10].

The role of DRAM in downregulation of different proteins is not yet known, but its structure suggests it is a small hydrophobic protein with six transmembrane domains [30], and it is very likely that DRAM is a regulated component in the process of fusion between lysosomes and some endosome vesicles. But since not all endosomal markers are degraded it means that the endosome population is heterogeneous, some are fused to the lysosomes, those containing VRK1, and others not, like the ones containing TSG101 which are implicated in vesicle recycling for downregulation of plasma membrane receptors [32], [41], [55]. DRAM might play two potentially different roles. One is to be the ligand for specific stable proteins that are removed by autophagy, which we consider unlikely since we could not detect a direct interaction between VRK1 and DRAM. Alternatively, DRAM might function as a marker of a subpopulation of vesicles destined for fusion to lysosomes and thus identified endosomal vesicles; in this context DRAM might be a receptor for a lysosomal protein required for vesicle fusion. The small size and the six transmembrane domains of DRAM suggests that most of it is not exposed, that is more logical to think of it as interacting with an unknown protein with lysosomes or an intermediate vesicle in the autophagic pathway, rather than a receptor for specific proteins destined for degradation in this pathway. Downregulation of DRAM in human cancer, like in melanomas [56], not only permits the survival of stress damaged cells, but also contributes to the maintenance of proteins which promote progression of the cell cycle, as is the case of VRK1, implicated in early G0/G1 [13] resulting in a mitotic delay [57], and required for Golgi fragmentation in mitosis [39], but which is likely to have additional roles along the cell cycle. In cells resistant to carboplatin there is an increase in VRK1 levels, probably reflecting a defective p53 response, and also favoring cell survival [58].

The accumulation of VRK1 is likely to drive to progression of cell division and thus expand a cell population that is mutated and implicated in tumor development. In tumors there are two possible situations resulting in VRK1 accumulation. Tumors with mutations in p53 will not induce DRAM and thus result in VRK1 accumulation. This correlation has already been detected in lung carcinomas [25] and in breast cancer (unpublished results). Alternatively, if there were tumors with DRAM inactivating mutations [40], they should also accumulate VRK1, but this possibility has not yet been studied. It is important to note that in tumors with p53 mutations, which are unable to induce DRAM expression, there would be an alteration in the regulation of autophagy, and this defective response can contribute to genetic instability [59]. In addition, it has been reported that apoptosis and autophagy induced in response to stress and mediated by JNK activates DRAM expression [60], [61]. Interestingly, VRK1 phosphorylates c-Jun in the same residues, Ser63 and Ser73, as JNK and their phosphorylation has an additive effect [62], which suggests that cells might be responding to stress in two different ways that perhaps can cooperate in the regulation of the initial stress response. The removal of VRK1 could be considered as part of a rescue mechanism [63], and defective induction of autophagy can contribute to genomic instability, as shown in a breast cancer model [64]. Therefore, there must be a fine regulatory balance among different p53 mediated responses, such as the equilibrium between cell-cycle arrest, apoptosis and autophagy, for which there has to be an underlying equilibrium between regulation of protein accumulation and degradation to protect cells from accumulating DNA damage, once it has been repaired. These regulatory loops modulate the balance between cell cycle progression and cell death by apoptosis or autophagy in DNA damage responses.

## Materials and Methods

### Cell lines and tissue culture

The human fibroblast WS1 cell line (CRL-1502) was obtained from the ATCC (Manassas, VA). Cells were grown in DMEM with 10% fetal calf serum and supplemented with antibiotics and glutamine. H1299 lung carcinoma cell line was grown in RPMI with 10% fetal calf serum and supplemented with antibiotics and glutamine (CRL-5803, ATCC) [13], [23], [24]. Cells plated on 100 mm dishes were treated with UV-C (254 nm) light in a Stratalinker. The real value of the UV dose delivered in each the experiment was confirmed by direct internal measurement with a radiometer Spectroline XS-254nm-UVC (Spectronics Corporation, Westbury, NY). Alternatively cells were treated with different drugs (Doxorubicin 0.5 µM, Etoposide 20 µM, Leptomycin B 0.2 nM [36] or IR (9 Gy) for WS1, or transfected for H1299.

### Plasmids and transfections

Plasmid pCB6+p53 were from K. Vousden (The Beatson Institute for Cancer Research, Glasgow, Scotland, UK) and pCMV-p53^R175H^, pCMV-p53^R248W^ and pCMV-p53^R273H^ were from B. Vogelstein (John Hopkins University). Plasmid pCDNA3-DRAM-Myc-His was a gift from K.M. Ryan [30]. VRK1, VRK2A and VRK2B were expressed from plasmids pCEFL-HA-VRK1, pCEFL-HA-VRK1(K179E) [18], [23], pCEFL-HA-VRK2A and pCEFL-HA-VRK2B [34]. TSG101 was expressed from plasmid pHA-TSG101 [65]. In all transfection experiments the total amount of DNA was always kept constant with empty vector.

### Knock-down of VRK1, DRAM, and Beclin-1

Synthetic SMART specific siRNA duplexes were purchased from Dharmacon RNA Technologies (Lafayette, CO). The targeted sequence for human DRAM (Accession number: NM_018370) was knock-down using siRNA (siDRAM-01 from Dharmacon RNAi Technologies); sense sequence: 5- CCACAGAAAUCAAUGGUGAUU; antisense sequence: 5′-P-UCACCAUUGAUUUCUGUGGUU. The targeted sequence for VRK1 (Accession number: NM_003384) was CAAGGAACCTGGTGTTGAA (duplex siVRK1-2) from Dharmacon. Beclin-1 was knocked down with siRNA-BECN1 on-Target plus SMART pool from Dharmacon. Functional siCONTROL non-targeting siRNA pool from Dharmacon was used as a negative control and fluorescently labeled siGLO Lamin A/C siRNA for transfection efficiency. Transfections of siRNA duplexes at 100–200 nM final concentration were carried out using Lipofectamine 2000 reagent (Invitrogen, Carlsbad, CA) following manufacturer instructions. After transfection, cells were processed for western blot or qRT-PCR as previously reported [13], [23].

### qRT-PCR

WS1 cells were washed in ice-cold PBS. Total RNA was extracted using the “RNAeasy extraction kit” from Quiagen (Hilden, Germany). RNA was analyzed and quantified using a Bioanalyzer 2100 nano-lab chip from Agilent Technologies (Böblingen, Germany). 100 ng of total RNA were used in a one-step reverse transcription real-time PCR amplification reaction using the “Quantitec SYBR Green RT-PCR kit” from Qiagen in an iCycler (BioRad, Hercules, CA). The reaction was analyzed with iCycler software (BioRad) and PCR products were resolved in a 1.5% agarose ethidium-bromide gel. The following forward (F) and reverse (R) primers were used for specific human DRAM message detection. Human DRAM (DRAM-F: 5′- TCAAATATCACCATTGATTTCTGT -3′; DRAM-R: 5′- GCCACATACGGATGGTCATCTCTG -3′) [30], human VRK1 (VRK1-F: 5′-CCAACGAGCTGCAAAACC-3′; VRK1-R: 5′-TGTCATGTAGACCAGACCCCC-3′) [13], and GAPDH amplification was used as internal control (GAPDH-F: 5′-GGTCTTACTCCTTGGAGGCCATGT-3′; GAPDH-R: 5′-ACCTAACTACATGGTTTACATGTT-3′) [13].

### Antibodies and immunoblots analysis

Cell extracts were prepared by homogenization in lysis buffer (Tris-HCl 50 mM pH 8, 200 mM NaCl, 5 mM EDTA, 1%Triton X-100 and protease and phosphatase inhibitors), and 40 micrograms were loaded for each sample in a 10% SDS-PAGE and transferred to Immobilon-P membrane (Millipore, Billerica, MA); membranes were processed for western blot, detection with a chemiluminescence ECL kit (GE Healthcare) that were quantified in the linear response range using an FX Personal Imager (BioRad) as previously reported [24].

Human VRK1 protein was detected with the 1F6 or 1B5 monoclonal antibodies [38]. The p53 protein was detected with a mixture of DO1 antibody from Santa Cruz Biotechnology (Santa Cruz, CA) and Pab1801 (Santa Cruz, CA) used at 1∶500 and 1∶1000 respectively. To detect endogenous p53 phosphorylated in Thr18 was detected with a rabbit polyclonal antibody from Cell Signaling Technology [18]. DRAM was detected with an antibody from ABCAM (Cambridge, UK) or myc epitope (Anti-Myc Tag Polyclonal Antibody, Upstate).VRK2A/B and TSG101 were detected with an anti HA epitope monoclonal antibody (Covance, Emeryville, CA). Endogenous TSG101 was detected with a rabbit polyclonal antibody [65]. The following antibodies were used: EEA1 detected with mAb 4/EEA1, and GM130 with mAb 35/GM130 from BD Transduction Laboratories (San José, CA). Giantin detected with polyclonal PRB-114C from Covance. LAMP2 detected with mAb H4B4, Beclin-1 was detected with a polyclonal antibody (sc-11427), p21 with mAb F-5 (sc-6246), and p62/SQSTM1 was detected with a monoclonal antibody (sc-28359), all from Santa Cruz Biotechnology (Santa Cruz, CA). LC3B was detected with a polyclonal antibody from Cell Signaling. Caveolin was detected with a rabbit polyclonal antibody from BD Biosciences. Hdm2 was detected with a monoclonal mouse Anti-Human MDM2 protein (Clone SMP14) from DAKO. All the antibodies were used at a 1∶1000 dilution for western blots, and at1∶100 for immunofluorescence. β-actin was detected with monoclonal antibody (Clone AC-15) from Sigma (St. Louis, MO) at a 1∶5000 dilution.

### Immunofluorescence and confocal microscopy

H1299 cells (5×10^5^) were plated on 10-cm^2^ dishes containing 1-cm-diameter sterile glass cover slips. The coverslips were stained twenty-four hours after DRAM transfection with specific antibodies for the endogenous and transfected proteins. Cells were washed three times with PBS and then fixed in 4% paraformaldehyde in PBS for 30 min at room temperature. After fixation the cells were permeabilized in cold PBS containing 0.2% Triton X-100 for 30 min and then treated with glycine 10 mM for 10 min at room temperature. Staining with antibodies was as previously reported [18], [39]. Subcelular localization was analyzed with a Zeiss LSM 510 confocal microscope.

## Supporting Information

Figure S1 **Dose dependent effect DRAM on levels of VRK2A (A), VRK2B (B).** H1299 cells were transfected with a fixed amount of VRK plasmids, and varying concentrations of DRAM. Plasmid pCDNA3-DRAM-Myc-His (Crighton et al., 2006). Plasmids pCEFL-HA-VRK2A o pCEFL-HA-VRK2B has been previously reported (Blanco, et al. 2006). To the bottom is shown the quantification of corresponding immunoblots. VRK proteins were detected with an anti HA antibody. DRAM was detected with an anti myc antibody. (C). Overexpression of p53 (left) or DRAM (right) induce downregulation of kinase-dead VRK1(K179E).
(TIF)

Figure S2 **DNA damage does not affect the effectiveness of BECN1 knockdown.** The knockdown of beclin-1 using siBECN1 is not affected by the time after induction of DNA damage by UV light. The experiment was performed in WS1 cells. The antibodies used in the immunoblots are described in the Materials and Methods section.
(TIF)
