# Supplementary figures and images for: Downregulation of VRK1 by p53 in Response to DNA Damage Is Mediated by the Autophagic Pathway
Source: PLoS One. 2011 Feb 28;6(2):e17320. doi: 10.1371/journal.pone.0017320 (PMC3046209; doi:10.1371/journal.pone.0017320)

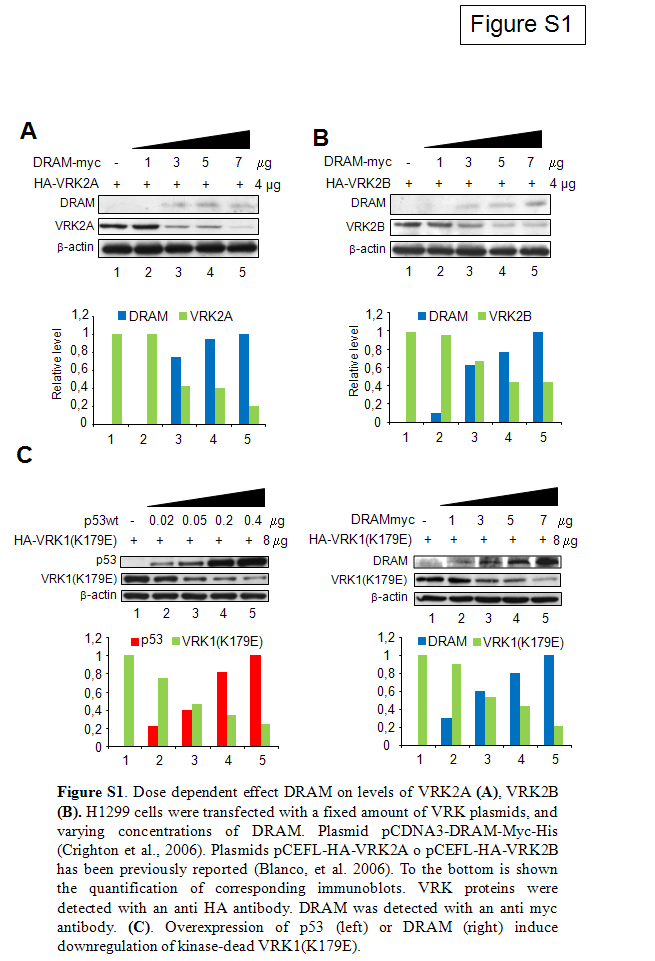

Supplement: Figure S1 — Dose dependent effect DRAM on levels of VRK2A (A), VRK2B (B). H1299 cells were transfected with a fixed amount of VRK plasmids, and varying concentrations of DRAM. Plasmid pCDNA3-DRAM-Myc-His (Crighton et al., 2006). Plasmids pCEFL-HA-VRK2A o pCEFL-HA-VRK2B has been previously reported (Blanco, et al. 2006). To the bottom is shown the quantification of corresponding immunoblots. VRK proteins were detected with an anti HA antibody. DRAM was detected with an anti myc antibody. (C). Overexpression of p53 (left) or DRAM (right) induce downregulation of kinase-dead VRK1(K179E). (TIF) [file pone.0017320.s001.tif]

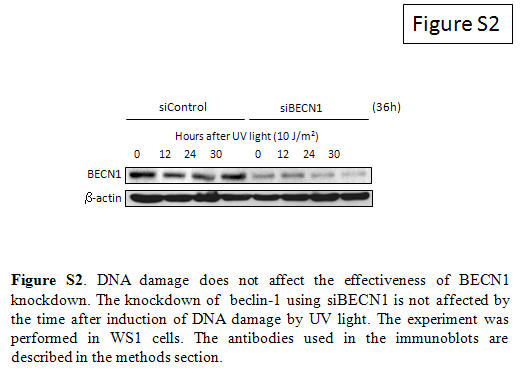

Supplement: Figure S2 — DNA damage does not affect the effectiveness of BECN1 knockdown. The knockdown of beclin-1 using siBECN1 is not affected by the time after induction of DNA damage by UV light. The experiment was performed in WS1 cells. The antibodies used in the immunoblots are described in the Materials and Methods section. (TIF) [file pone.0017320.s002.tif]
